# Reduction of Health Care-Associated Infections (HAIs) with Antimicrobial Inorganic Nanoparticles Incorporated in Medical Textiles: An Economic Assessment

**DOI:** 10.3390/nano10050999

**Published:** 2020-05-23

**Authors:** Finbarr Murphy, Anat Tchetchik, Irini Furxhi

**Affiliations:** 1 Transgero Limited, Naomh Treasa, Cullinagh, Newcastle West, Co., V42 V384 Limerick, Ireland; irini.furxhi@transgero.eu; 2 Kemmy Business School, University of Limerick, V94 PH93 Limerick, Ireland; 3 Department of Geography and Environment, Bar-Ilan University, 5290002 Ramat-Gan, Israel; Anat.Tchetchik@biu.ac.il

**Keywords:** nanotextiles, mamomaterials, antimicrobial

## Abstract

Health care-associated infections (HAIs) affect millions of patients annually with up to 80,000 affected in Europe on any given day. This represents a significant societal and economic burden. Staff training, hand hygiene, patient identification and isolation and controlled antibiotic use are some of the standard ways to reduce HAI incidence but this is time consuming and subject and subject to rigorous implementation. In addition, the lack of antimicrobial activity of some disinfectants against healthcare-associated pathogens may also affect the efficacy of disinfection practices. Textiles are an attractive substrate for pathogens because of contact with the human body with the attendant warmth and moisture. Textiles and surfaces coated with engineered nanomaterials (ENMs) have shown considerable promise in reducing the microbial burden on those surfaces. Studies have also shown that this antimicrobial affect can reduce the incidence of HAIs. For all of the promising research, there has been an absence of study on the economic effectiveness of ENM coated materials in a healthcare setting. This article examines the relative economic efficacy of ENM coated materials against an antiseptic approach. The goal is to establish the economic efficacy of the widespread usage of ENM coated materials in a healthcare setting. In the absence of detailed and segregated costs, benefits and control variables over at least cross sectional data or time series, an aggregated approach is warranted. This approach, while relying on some supposition allows for a comparison with similar data regarding standard treatment to reduce HAIs and provides a reasonable economic comparison. We find that while, relative to antiseptics, ENM coated textiles represent a significant clinical advantage, they can also offer considerable cost savings.

## 1. Introduction

Healthcare associated infections (HAIs) are infections that acquired while receiving health care in a hospital or other health care facility that first appear 48 h or more after hospital admission [1] or within 30 days after discharge following in patientcare [2]. In the US, is has been reported that HAIs are the most common complications of hospital care and one of the top 10 causes of death [3]. The overall direct cost of HAIs to hospitals in the US ranges from USD 28 billion to 45 billion [4]. In the EU, it is estimated that 6.5% of patients in acute care hospitals had at least one HAI [5]. Hospital related costs in Europe are estimated at USD 12 billion per year [6]. In Australia the burden of HAI has been calculated to be approximately 165,000 cases per year, rendering them the most common complication for hospital patients [7,8]. Given their huge costs, the World Health Organisation has been leading efforts to formulate and publish Clinical guidelines and interventions in an effort to decrease the incidence of HAIs [9].

The most common sources for HAIs are person-to-person transmission, medical equipment or devices, ventilator-associated pneumonia [10], healthcare personnel, contaminated drugs and food. Medical textiles are considered one of the possible vehicles of transmission [11]. To address this risk, current guidelines by the Centre for Disease Control and Prevention (CDC) include appropriate washing of hospital textiles (i.e., collecting, sorting, transporting, laundering with appropriate water temperature, type of detergents, disinfectant, rinsing and finishing, drying and ironing), but also their disinfection and in certain cases even sterilization. These procedures are effective but subject to continuous and rigorous implementation. Moreover, there are evidence of microorganisms survival on medical hospital textiles after laundering [12], though the literature in the field is confusing and even contradictory. Against this backdrop, the use of antimicrobial inorganic ENMs incorporated in medical textiles is among the most promising strategies to provide aseptic conditions in hospitals [13]. Some engineered nanomaterials (ENMs) have proven anti-microbial properties that are particularly effective at the nano scale in contrast to their bulk form ENMs. In particular copper and silver oxide nanoparticles and nanoclays, are already used in food packaging [14] and food preservation [15]. The anti-microbial properties of some ENMs have encouraged significant research in their application in health science. In contemporary settings, ENMs have found multitudinous applications in healthcare environments to reduce the incidence of infections. In that vein, instances of ENMs incorporated into medical textile has grown significantly over the last decade. In order to justify the use of ENMs embedded medical textiles and to achieve a large scale implementation of them, a benefit-cost analysis is warranted. Arguably, the application of ENMs embedded medical textiles should not only prove economically justifiable, it also needs to comparable to in terms of (cost) effectiveness to traditional routines to reduce the incidence of infections.

Traditional routines to reduce the incidence of infections include thorough cleaning using health care antiseptics, personnel hand washes and antiseptics, surgical hand scrubs, patient preoperative and preinjection skin preparations, etc. These are widely regarded as the most effective means to reduce HAI incidence. However, evidence shows that effective cleaning is not always implemented. In many studies, researchers marked high-contact surfaces with a ultraviolet marker to determine if those surface areas were subsequently cleaned [16,17]. Carling, Briggs, Perkins, Highlander [18] found that after routine cleaning only 47% of the surfaces had actually been cleaned sufficiently. Moreover, there seems to be a lack of compliance with hand hygiene protocols [19]. The gold standard method for monitoring hand hygiene compliance is direct observation however this audit method is less than optimal. Studies have pointed out the advantages of electronic monitoring systems, yet, their cost effectiveness has not been determined. Armellino et al. [20] describe a cost of USD 50,000 for the installation of an electronic monitoring system for a 17 bed intensive care unit yet, they do not report the cost associated with ongoing real-time feedback.

The conventional approach to HAI reduction has, arguably, reached something of a plateau. This suggests that antimicrobial ENM coated surfaces can represent an orthogonal approach with significant promise. ENMs can be used on hard surfaces but also on upholstery and fabrics. The active ingredient is usually copper nanoparticles (CuNP) and silver nanoparticles (AgNP) although there are other options such as Zinc oxide nanoparticles (ZnNP). These nanoparticles kill pathogens when they oxidize and release ions, which enter the bacteria membrane and destroy the pathogen. Metal nanoparticles are more powerful than their bulk form because their large surface area relative to their mass increases the number of ions released. Furthermore, the nanoparticles can continue to reduce the microbial burden almost perpetually [21].

We focus on textiles as they are an excellent substrate for bacterial proliferation and textiles are considered a transmission vehicle [11]. This is particularly true when in contact with human skin. The natural secretions and desquamation provide the excellent moisture and temperature conditions for pathogens. The textile itself has a very large surface area, again, providing an excellent environment for bioburdens. Studies have shown that healthcare fabrics (sheets, pyjamas, uniforms, etc.) can contain heave microbial burdens [22,23,24]. Indeed, pathogens can survive for weeks on textiles and even after an industrial wash cycle [24,25].

Another reason for our focus on textiles is that their properties as an ideal substrate for pathogens make them also difficult to clean using traditional antiseptics except through a conventional wash cycle. Indeed, textiles such as uniforms, allow the pathogens to be transported from patient to patient. Similarly, movement of textiles such as bedsheets and curtains can aerosolize the pathogens present on the fabric. All told, ENM coated textiles represent a discrete new front in HAI reduction strategies.

It is clear that a multifaceted approach to HAI reduction that includes training, procedures, health care antiseptics, hydrogen peroxide vapour, and ultraviolet light along with self-cleaning surfaced using ENMs is required [26]. However, there is a trade-off between a reduction of HAIs and the allocation of finite resources to implement those reductions. Both the health care antiseptic and ENM coated textile markets are witnessing significant growth period however their absolute and relative performance are largely undetermined due to the fact that, there are currently no randomized controlled trials of the efficacy of these systems in preventing HAIs [27]. Given this deficiency, some studies offer a cost effective/benefit approach to guide decision making [28,29]. In this article, we aim at closing a gap in current knowledge by specifically examining the economic efficacy of using antimicrobial ENMs incorporated in medical textiles. More specifically, we compare the economic advantage of health care antiseptics with that of textiles coated with ENMs. To be clear, we are not supposing that a binary choice should be made between health care antiseptics and textiles coated with ENMs, it is safe to assume that these two methodologies are complementary in their applications. Yet, this research does provide guidance on the difficult choice faced by health care administrators and clinicians in allocation economic resources to reduce incidence of HAIs, by examining to which extent textiles coated with ENMs can be considered at a level playing field with health care antiseptics. Due to a lack of detailed data at a ward level, we use an aggregate data framework, a modified approach of Schmier et al. [30].

The objective of this article is to gauge the economic benefits afforded by the use of ENM coated textiles. A stand alone cost benefit analysis is not practical because there is insufficient data to make a determination. We use a relative comparison analysis to compare the economic effectiveness of ENM coated materials against a standard antiseptic approach. The results can assist in the administrative decision to utilize ENM costed textiles in healthcare settings.

For the remainder of this article, we first examine the relevance of HAIs and their economic consequences, particularly in Europe. We then examine the prevalence and use, cost and effectiveness of both antiseptics and ENM coated textiles to set up a comparison. The next section introduces our methodology. This is followed by detailed results and an associated discussion. Before offering a conclusion, we offer some insights on how a regulatory approach would impact the current environment.

## 2. Healthcare-Associated Infections (HAIs)

Healthcare-associated infections (HAIs) are defined as infections occurring in a patient during the process of care in a hospital or other health care facility which was not present or incubating at the time of admission [31]. HAIs also referred to as “nosocomial” or “hospital” infections. The European Centre for Disease Prevention and Control (ECDC) estimate that, on any given day, about 80,000 patients, or one in 18 patients, in European hospitals have at least one healthcare-associated infection [32]. The most frequent types of HAI are surgical site infections, urinary tract infections, pneumonia, bloodstream infections and gastrointestinal infections. About 7% of patients in acute care hospitals in Europe experience a HAI. The documented range of HAI’s per patient is 3–10% across EU member states with an average rate of 7% [33]. MRSA (Methicillin-resistant Staphylococcus aureus) infections are the most common and increase the hospitalization period from an average of 4 to 14 days with additional costs varying from EUR 10,000 to EUR 36,000 per patient [34,35]. The annual mortality directly attributable to nosocomial infections is estimated to be about 37,000 in the EU but this number could increase to 110,000 if indirect deaths are counted [33].

There is a clear evidence between the incidence of HAIs and the environmental microbial contamination present in health care setting [36,37]. Furthermore, common nosocomial pathogens may persist on surfaces for months providing a continuous source of microbial transmission [24,38]. These hard and soft surfaces in clinical settings serve as a persistent source for nosocomial pathogens, that can be transmitted through contact and aerosolized particles with either direct transmission (patient touching contaminated surfaces) or indirect (hospital personnel may contaminate themselves by touching contaminated surfaces and subsequently transfer the microorganisms to the patients).

Aggressive measures have been introduced over the past several years to reduce the incidence of HAI. These measures include improved staff training, better disinfection and hygiene regimes and patient monitoring and isolation of infected patients. Specialist infection control staff, disposable equipment and antibiotic control programmes have also contributed. Despite these improvements, it appears that the conventional approach to HAI reduction has reached something of a plateau and there is a need to consider the use of additional measures and modern technologies to reduce patient hospitalization and concurrent costs. New technologies fall into several categories such as: (1) new liquid surface disinfectants (e.g., improved hydrogen peroxide liquid disinfectants), (2) improved methods for applying disinfectants (e.g., Microfiber cloths or mops and ultra-microfiber cloths for applying liquid disinfectants to surfaces), (3) self-disinfecting surfaces (e.g., coating surfaces with heavy metals such as copper or silver), (4) light-activated photosensitizers (e.g., nanosized titanium dioxide to surfaces and using UV light to generate reactive oxygen species that can disinfect surfaces), and (5) no-touch (automated) technologies (e.g., aerosolized hydrogen peroxide, hydrogen peroxide vapor systems, gaseous ozone, chlorine dioxide, saturated steam systems) [27].

Among the third category, active antimicrobial surfaces and fabrics represent a novel and effective approach to reducing the bacterial burden in clinical settings, especially those surrounding the patients. In particular, within medical environments, surfaces containing copper or copper oxide and silver have been found to reduce bioburden and the transmission of nosocomial pathogens [39,40,41,42,43].

Despite the enthusiasm and potential for ENM coated surfaces, particularly textiles, a significant understanding of the relative cost effectiveness of ENM coated surfaces is elusive. That is, ENM coated textiles have a demonstrated capacity to reduce the incidence of HAIs but this comes at an economic cost which is not necessarily justified when compared to existing HAI reduction techniques. To better answer this question, in this article, we examine the economic benefits of deploying ENM coated textiles against the use of health care antiseptics. The answer will allow healthcare administrators better allocate funding resources to optimize HAI incidence.

### 2.1. Health Care Antiseptics

The global antiseptics and disinfectants market size was valued at USD 16.75 billion in 2018 and is expected to grow to USD 28.1 billion by 2026, an CAGR of 6.7% [44]. The high incidence of HAIs and their consequent costs and the increasing awareness of hygiene is driving demand. Medical device disinfectants, enzymatic cleaners and surface disinfectants are the three main types of prevailing disinfectants. Yet, evaluating their efficacy in reducing HIA cases and costs is challenging.

Schmier et al. [30] provide interesting research on the economic savings associated with the use of hospital antiseptic products on preventable HAIs. In their paper, they show that antiseptic usage reduces HAI incidence and shows a cost saving of up to USD 4.25 million annually in the US alone. Schimier et al. use a simple approach that first examines the total number of HAI’s, reduces that by the amount of preventable HAI’s and reduces that further by those HAI’s prevented by antiseptics.

Using antiseptic as a baseline HAI control means, we employ a modified version of Schmier et al.’s (2018) approach to determine the HAI reduction achieved by ENM coated hospital textiles. 

### 2.2. ENM Coated Hospital Textiles

The global value of the medical textile market was valued at USD 12.2 billion in 2018 and is expected to reach USD 18.5 billion by 2025 [45]. Medical textiles can include surgical gowns, gloves, drapes, facemasks, dresses, and linens, which could be disposable or reusable based on the use-case. Overcash [46] shows that factors such as cost, protection, and comfort are reasonably similar between disposable and reusable gowns and drapes but reusable surgical textiles offer increased sustainability benefits over similar disposable products in energy (200–300%), water (250–330%), carbon footprint (200–300%), volatile organics, solid wastes (750%), and instrument recovery.

Antimicrobial textiles typically use copper, silver or a combination as the active agent though Zinc Oxide has also proven effective [47] in a burns unit. Table 1 below shows a summary of some of the research on HAI reduction by antimicrobial textiles in a healthcare setting.

It should be noted that Madden, Heon, Sifri [53] showed no significant reduction in incidences of healthcare facility-onset Clostridium difficile infection or MDRO acquisition when copper impregnated linen was used in a 40-bed long-term acute-care hospital.

Reducing the surface bioburden is an effective strategy to reduce HAIs [39,40,41]. The inherent biocidal properties of substances such as silver and copper surfaces offer an advantage to conventional cleaning, as the effect is continuous rather than episodic. For example, [40] showed a 58% reduction in the rate of HAI when patients stayed in intensive care unit (ICU) with copper alloy surfaces.

In addition to copper oxide particles, silver nanoparticle (AgNP) and AgNP/reduced graphene oxide (rGO) nanocomposite demonstrate antimicrobial activity against HAIs. [54]

## 3. Methods

Our approach uses a modified version of the methodology proposed by Schmier et al. [30] where they propose an antiseptic cost-benefit analysis. They identified five HAIs of interest (catheter-associated urinary tract infections, central line-associated bloodstream infections, gastrointestinal infections caused by Clostridium difficile, hospital-or ventilator-associated pneumonia, and surgical site infections). They then employ four initial inputs. First, the national estimates of the number of cases of each type of HAI. Second, the proportion of those HAIs that are preventable. Third, the proportion of preventable HAIs that can be prevented by the use of antiseptic or disinfectant. Fourth and finally, the average hospital cost incurred by each HAI. They gather these figures from published literature and aggregate the data. Their final result estimates the range of low and high estimates of annual HAI cases avoided through use of health care antiseptics at 12,100 and 223,000 respectively. Their estimate of the associated economic costs is USD 142 million and USD 4.25 billion, respectively.

One drawback of this approach is that the cost of the antiseptics used and the cost of their application is not included in the cost benefit analysis. Another drawback is the evident scale of the uncertainty in the results as well as the inability to control for other factors that could affect HIA prevalence and avoidance. We develop this model by estimating the cost associated with the purchase and application of the antiseptic solution. We then apply a similar approach for the purchase and use of ENM coated textiles. Whilst the cost-benefit analysis of both antiseptics and ENM coated textiles contain high uncertainty, by comparing the relative cost-benefit analysis, we argue that the respective variability is less concerning.

*H* × *P* × *SP_T_* × *B_T_* − *C_T_* (1)

Equation (1) above shows the Cost Benefit model of antiseptics. *H* is the annual total number of HAI incidence in the EU, *P* is portion of the preventable number of HAIs and *SP_T_* is the portion of preventable HAIs, due to antiseptics. *B_T_* is the average economic cost of a patient suffering a HAI event. Finally, *C_T_* is the cost of the antiseptic treatment, which includes the purchase and application of the antiseptic.

*H* × *P* × *SP_E_* × *B_T_* − *C_E_* (2)

Equation (2) above shows the same Cost Benefit model but applying ENM coated textiles as the unit of analysis. *SP_E_* is the portion of preventable HAIs due to ENM coated textiles and *C_E_* is the cost of the ENM coated textiles.

Finally while Schmier et al. [30] use US data, this article uses EU data. Aggregate incidence rates of HAI in the EU are similar to those in the US and, to an extent, can be used as a proxy for results in the US and vis a versa.

## 4. Results and Discussion

### 4.1. Total Number of HAIs

In 2016 and 2017, the European Centre for Disease Control (ECDC) coordinated site surveys to collect data on HAIs in hospitals and long-term care facilities in EU/EEA countries [5]. Depending on the nature of the HAI, the infection can be mild or severe and increases patient hospitalization and hospital costs. HAIs in hospitals alone cause more deaths in Europe than any other infectious disease under surveillance at ECDC.

A total of 8.8 million HAIs were estimated to occur each year in European hospitals and long-term care facilities combined. HAIs in hospitals (for example pneumonia, surgical site infections and bloodstream infections, are usually more severe than HAIs in long-term care facilities (for example respiratory infections other than pneumonia, urinary tract infections and skin and soft issue infections).

### 4.2. Proportion of HAIs That Are Preventable

An important economic question is whether zero incidents of HAIs is an optimal solution. According to a positive economic perspective, equating marginal utility with marginal cost incurred by expenditure to prevent HAIs, may not result with zero HAIs being the social optimum. However, it appears that even if zero incidents of HAIs were an optimal solution, preventing all HAIs is not possible. According to Umscheid et al. [55] between 55% and 70% are reasonably preventable which would equate to between 1.1 and 1.4 million avoidable infections and 49,500–63,000 avoidable deaths annually in the US. The financial costs associated with these potentially preventable infections are estimated to be as high as USD 23 billion per annum. Harbarth et al. [56] reviewed 30 reports and estimated that 20% of HAIs are preventable but in their review found that between 10% and 70% prevention rates are reported. More recently, Gastmeier et al. [57] report that 20% to 30% of HAIs in Germany could be preventable primarily through improved adherence to hygiene recommendations and optimisation of procedures. In either case, the implementation of comprehensive, evidence-based prevention strategies will tend to increase the prevention of HAIs but since resources are limited, an economic guideline needs to be utilized in order to optimally allocate scarce resources

Clearly, the proportion of HAIs that are preventable is context specific, and depends on the infection type and infection location along with the application of prevention procedures. In this study we use a range of values, 15%, 25% and 35% as appropriately conservative prevention estimates.

### 4.3. Prevented Cases Due to Antiseptics

We are not aware of research that specifies prevention by antiseptic and by pathogen. We further assume aggregate prevention based on overall incidences of HAI rather than specific pathogen types. Several studies, notably Gordin et al. [58], examine the effectiveness of disinfectants on HAI pathogens. Aboualizadeh et al. [59] showed the effectiveness of disinfectants, namely ethanol, isopropanol, sodium hypochlorite, triclosan and triclocarban on MSRA pathogens. These (and many other studies) show the high effectiveness of antiseptic cleansers. On the other hand, the application of the disinfectants by hospital staff can be less than optimal. This is most clearly evidenced by [60] paper on the effectiveness of a hospital-wide programme to improve compliance with hand hygiene.

In line with Schmier et al. [30], as a proxy for prevented cases due to antiseptics, we use a range of values of 10%, 20%, and 30%, not based on specific study but a conservative reflection of estimates in the literature.

### 4.4. Prevented Cases Due to ENM Coated Textiles

Copper oxide (CuO) impregnated linens are the most common anti-microbial textile deployed in health care setting though Silver Oxide (AgO), Zinc Oxide (ZnO) are also used. The additional ions of the ENM combine bind with the microbes that come in contact with them and disrupt their normal cell function, ultimately destroying the pathogen. Table 1 highlights some research on the effectiveness of ENM coated textiles in reducing HAI incidence. In some research, the efficacy of ENM costed textiles in reducing HAI incidence is explored in general while some research examines the reduction of specific pathogens on the textile surface.

Based on Table 1, we submit a HAI reduction rate of 20%, 30% and 40%. This is deemed judiciously conservative based on a survey of extant research. A complicating factor for (multiuse) nano-textiles is the wash cycle. Fabrics, textiles, and clothing used in health-care settings are disinfected and hygienically cleaned but not sterilised during laundering. Laundering cycles consist of flushing, main wash, bleaching, rinsing, and souring. Hot water washing is typically done at 75C. Clean, wet textiles are then dried, pressed, folded and packaged for redistribution back to the facility.

Multiple wash cycles can reduce the antimicrobial capacity of the active metal; however, this is largely overcome by the metal silver deposition using ultrasound irradiation. In this method, nanoparticles thrown to the fabric’s surface by sonochemical microjets are high enough to cause melting and carbonization with the of the textile fibers. Using this method, Perelshtein et al. [61] showed the material staying on the fabric for at least 20 washing cycles without a reduction in the (silver) content. Given the effectiveness of the coating process and the conservative estimates of the HAI reduction rates by ENM coated materials, we feel the reduction of the efficacy of the antibacterial coating though the washing cycle can be accommodated in the existing estimates.

### 4.5. Costs of Antiseptics

The global antiseptics and disinfectants market size was valued at USD 16.75 billion [44]. In 2016, the world spent USD 7.5 trillion on health, representing close to 10% of global GDP [62]. General government expenditure in the EU on health amounted to EUR 1080 billion or 7.0 % of GDP in 2017 [63]. Using a simple ratio, this suggests the antiseptic market in the EU is valued at EUR EUR 3 billion per annum.

This estimate does not include the cost of application of the antiseptics which is likely to be a multiple of the purchase cost. The difficulty here lies in determining the costs of applying antiseptics. The task is performed by a large variety or healthcare personal from administrative staff (procurement), to facility distributors, cleaning staff and medical staff. For the most part, the use of antiseptics is an integral part of day-to-day patient care. Nevertheless, a reasonable assumption is that the application of antiseptics is double the cost of purchase at EUR 6 billion.

### 4.6. Cost of ENM Coated Textiles

The global value of the medical textile market was valued at USD 12.2 billion in 2018 and is expected to reach USD 18.5 billion by 2025 [45]. Using the ratio of EU healthcare spend to the global spend, suggests that the EU spends EUR 3 billion per annum on medical textiles. The global Antimicrobial textile market is estimated to grow from USD 9.5 Billion in 2019 to USD 12.3 Billion in 2024, at a CAGR of 5.4% [64] however, these estimates include general hygiene products, not just healthcare textiles.

Using a websearch, at the time of writing, we estimate the unit cost of a high-end commercially bed sheet at EUR 15 per meter squared. Additionally, we estimate the cost of coating this unit at EUR 0.30 per unit or a 2% increase in cost. The overall medical textile market includes gowns, sheets, protective wear and these products come in a variety of fabrics. However, assuming the overall EU medical textile market is valued at EUR 3 billion, this mean the additional cost of coating the textiles with antimicrobial metals is approximately EUR 60 million (per annum).

### 4.7. HAI Economic Costs

HAI in Europe is responsible for approximately 16 million extra days spent in hospital per year and a quarter of all adverse events suffered by hospital patients. This amounts to an estimated direct cost of EUR 7 billion [65]. These costs do not include the indirect costs of lost earnings, reduced work productivity, short-and long-term morbidity and mortality or time and costs spent on hospital visits. Equally, it does not include the intangible costs of pain and suffering, or changes in life quality. In the US, Scott [66] estimates direct costs at US USD 6.5 billion while Stone [4] estimates US indirect costs between USD 28 billion to 45 billion, which entail a ratio of 4.3:1 between direct and indirect costs.

In our model, we assume a range of EU conservative direct costs only at EUR 7, EUR 12 and EUR 17 billion and accordingly indirect costs of EUR 30, EUR 52 and EUR 73 billion. In this study, we do not distinguish between HAI types which incur different costs. For example, pneumonia is more expensive to treat than a catheter-associated urinary tract infection. However, an aggregated total cost is sufficient to provide economic guidance.

### 4.8. Model Results

Based on our model, data from extant research and judicial assumptions, we find that the annual cost reduction of HAI instances in the EU by the use of antiseptics is between EUR 557 million and EUR 9474 million. These numbers are in line with similar research performed by Schmier et al. [30] for US data. Using the same approach, we find that the application of ENM coated (antimicrobial) textiles is between EUR 304 million and EUR 8038 million which is more than the antiseptic range.

More interestingly, we estimate the cost of purchasing and applying antiseptics to be about EUR 9 billion per annum in the EU while the cost of coating hospital textiles with an antimicrobial metal is only EUR 60 million per annum. This suggests that HAI reduction techniques and their associated cost savings should encompass ENM coated textiles as a standard tool to reduce infections.

### 4.9. Nanotoxicity

This study does not incorporate the toxicity potential of NPs embedded in the functional textiles. Extant literature indicates that Copper and Silver NPs result in several adverse effects such as reactive oxygen species generation, oxidative stress, inflammation, cytotoxicity, genotoxicity and immunotoxicity [67,68,69,70]. Physicochemical characteristics, such as particle shape, size, surface functionalization, the exposure dose, duration and mode are the main factors that define the toxicity of the nanoparticles [71]. A paucity of exposure data through different exposure routes such as oral ingestion or inhalation route and data lacunas regarding the key physicochemical properties that influence the toxicity of NPs, makes the integration of toxicity out of the scope of this study. A case-by-case hazard assessment of the nanomaterials is needed in each case to incorporate the toxicity into the economic evaluation. While a comprehensive understanding of the risks to patients and staff is some way off, the economic costs of those risks is difficult to gauge at the time of writing. One practical pathway would be to measure the increased costs, if any, to insurance premiums.

## 5. Conclusions

Health care-associated infections (HAIs) are a considerable economic burden to the global health sector directly costing USD 6.5 billion and additional USD 28 billion to 45 billion of indirect costs, in the US alone. These amounts ignore the societal costs and the incalculable suffering caused to victims of HAIs. In Europe, 7% of all admissions to acute hospitals result in a HAI with 37,000 direct fatalities and up to 110,000 fatalities if indirect deaths are included.

The current approach to combatting HAIs is pragmatic and focused on staff training, patient identification and isolation, use of antiseptics, antibiotic prescription monitoring and so on. Despite these measures, a flawed implementation and the nature of the pathogen suggests that the effectiveness of current methodologies may have plateaued.

ENMs have been considered to be the ‘‘material of the 21st century”. There is a significant body of research to show that surfaces ameliorated with ENM, particularly copper and silver oxides, can reduce the biological burden and therefore reduce the incidence of HAIs in health care facilities. The active agent of the coating is constant and perpetual. Textiles are a discrete surface type that are amenable to microbial persistence because of the nature of the material, the moisture and temperature conditions present and the very large surface area. These provide an ideal environment for microbial proliferation.

Our research shows that approximately similar amounts of money are spent on antiseptic and on hospital textiles per annum, namely EUR 3 billion. In examining the relative effectiveness of ENM coated textiles against antiseptics, we find that coating textiles with antibacterial ENMs is potential more effective than antiseptics. Furthermore, the additional cost of coating textiles with an ENM antibacterial substance is relatively inexpensive. In addition, coating textiles with an ENM does not require expansive monitoring. One potential impediment to the large scale roll-out of ENM coated textiles is the absence of a clear understanding of the human and environment risks posed by nanoparticles. These risks will negatively impact both the clinical and economic benefits afforded by ENM coated materials but it is not possible at this time to measure that impact.

To be clear, this is not an either/or situation. We use the effectiveness of antiseptics only as a means to judge the relative effectiveness of ENM coated textiles. We also acknowledge the relative paucity of information, the heterogeneous nature of the data and the suppositions (though conservative ones) made in our analysis. While our approach is not a rigorous economic analysis due to the lack of data, it is, nonetheless an important first step in determining the efficacy of ENM coated materials. Accordingly, our results and suggest a greater investment in ENM coated textiles is economically justified and certainly warrants further, detailed and comprehensive analysis. A note of caution is warranted given the current lack of knowledge regarding the safety in use of ENMs used in textile industry. If scientific evidence was sufficiently robust, regulators may mandate the use of ENM coated textile. Yet the current lack of knowledge suggests that some regulatory measures should be taken. These measure in turn, may increase the cost of these ENMs and thus question their cost effectiveness.

In this paper we have outlined the effectiveness of ENM coated textiles in reducing the incidence of HAIs. Despite this, there is a notable absence of wide scale usage of this technology in clinical settings. This is due, in part, to regulatory hurdles, human and environmental risk uncertainty and a paucity of clinical evidence. In our view, it will be necessary to provide a systematic test approach involving academic, clinical and commercial interests. The success, or otherwise, of these tests will ultimately be determined by the economic viability of the approach and, while we have determined that this is likely to be cost effective, the economic modelling needs to be included as part of future systematic testing.

## Figures and Tables

**Table 1:** Example papers showing a reduction of bioburden by antimicrobial textiles.

Authors	Result	Type
**[43]**	24% reduction in HAI per 1000 hospital days and a 27% savings in costs	Copper oxide impregnated linens
**[48]**	76% aggregate reduction in HAI	copper-impregnated composite hard surfaces and linens
**[49]**	29% reduction in antibiotic treatment initiation events (ATIEs)	copper oxide-impregnated textiles
**[50]**	28% reduction in total Clostridium difficile and multi drug resistant organisms MDRO.	copper-impregnated composite hard surfaces, bed linens and patient gowns
**[51]**	37% reduction of HAI Clostridium difficile and MDROs	copper oxide-impregnated linens
**[52]**	48% and 17% reduction of *Staphylococcus aureus* and *Escherichia coli*, respectively	Zinc Oxide (ZnO) nanoparticles with chitosan
